# Psychosis and the Control of Lucid Dreaming

**DOI:** 10.3389/fpsyg.2016.00294

**Published:** 2016-03-09

**Authors:** Natália B. Mota, Adara Resende, Sérgio A. Mota-Rolim, Mauro Copelli, Sidarta Ribeiro

**Affiliations:** ^1^Brain Institute, Federal University of Rio Grande do NorteNatal, Brazil; ^2^Onofre Lopes University Hospital, Federal University of Rio Grande do NorteNatal, Brazil; ^3^Physics Department, Federal University of PernambucoRecife, Brazil

**Keywords:** psychosis, schizophrenia, bipolar disorder, lucid dreams, dreaming

## Abstract

Dreaming and psychosis share important features, such as intrinsic sense perceptions independent of external stimulation, and a general lack of criticism that is associated with reduced frontal cerebral activity. Awareness of dreaming while a dream is happening defines lucid dreaming (LD), a state in which the prefrontal cortex is more active than during regular dreaming. For this reason, LD has been proposed to be potentially therapeutic for psychotic patients. According to this view, psychotic patients would be expected to report LD less frequently, and with lower control ability, than healthy subjects. Furthermore, psychotic patients able to experience LD should present milder psychiatric symptoms, in comparison with psychotic patients unable to experience LD. To test these hypotheses, we investigated LD features (occurrence, control abilities, frequency, and affective valence) and psychiatric symptoms (measure by PANSS, BPRS, and automated speech analysis) in 45 subjects with psychotic symptoms [25 with Schizophrenia (S) and 20 with Bipolar Disorder (B) diagnosis] versus 28 non-psychotic control (C) subjects. Psychotic lucid dreamers reported control of their dreams more frequently (67% of S and 73% of B) than non-psychotic lucid dreamers (only 23% of C; S > C with *p* = 0.0283, B > C with *p* = 0.0150). Importantly, there was no clinical advantage for lucid dreamers among psychotic patients, even for the diagnostic question specifically related to lack of judgment and insight. Despite some limitations (e.g., transversal design, large variation of medications), these preliminary results support the notion that LD is associated with psychosis, but falsify the hypotheses that we set out to test. A possible explanation is that psychosis enhances the experience of internal reality in detriment of external reality, and therefore lucid dreamers with psychotic symptoms would be more able to control their internal reality than non-psychotic lucid dreamers. Training dream lucidity is likely to produce safe psychological strengthening in a non-psychotic population, but in a psychotic population LD practice may further empower deliria and hallucinations, giving internal reality the appearance of external reality.

## Introduction

Dreaming and psychosis share important phenomenological and neurophysiological features (Gottesmann, 2005; Manoach and Stickgold, 2009; Mota-Rolim and Araujo, 2013; Dresler et al., 2014). In terms of subjective experience, both phenomena present intrinsic sense perceptions independent of external stimulation, associated with a lack of criticism (or rational judgment) regarding the bizarreness of these experiences (Cicogna and Bosinelli, 2001). The latter feature has been hypothesized to stem from the decrease in frontal cerebral activity that characterizes both psychosis and rapid-eye-movement (REM) sleep (Dresler et al., 2014; Voss et al., 2014). Yet, executive functions are not necessarily impaired during dreaming. It is possible to be aware of dreaming while a dream is happening, with partial or total control of the dream contents by the dreamer, a phenomenon called lucid dreaming (LD; Laberge et al., 1986; Mota-Rolim and Araujo, 2013; Stumbrys et al., 2013; Voss et al., 2014). Recent studies using functional magnetic resonance imaging (Dresler et al., 2012) and electroencephalography (Voss et al., 2009) indicate that LD is related to increased activity in the prefrontal cortex (Voss et al., 2009, 2014; Mota-Rolim et al., 2010; Neider et al., 2011; Dresler et al., 2012; Stumbrys et al., 2013). In agreement with this notion, transcranial electrical stimulation of the prefrontal cortex can induce dream awareness during REM sleep (Stumbrys et al., 2013; Voss et al., 2014). Frontal cortex activity correlates with self-consciousness, working memory, and attention (Postle, 2006). Therefore, an increase in frontal activity should contribute to lucidity during dreaming (Hobson, 2009; Voss et al., 2009), while a decrease in prefrontal activity should explain the lack of rational judgment in both psychosis and non-lucid dreams (Anticevic et al., 2012; Dresler et al., 2014).

Theories about human consciousness propose that the LD phenomenon is possible due to the linguistic ability of our species, which permits the semantic access of episodic memories of sensory origin (Edelman, 2003; Voss et al., 2013). By accessing episodic memories, the flow of thoughts can be reported, and the subjective ability of “mind wandering” can be shared with others. Similarly, dream mentation can be understood as spontaneous thinking, not associated to any external task (Fox et al., 2013). An important set of systems involved in this process is the default mode network (DMN), a functional circuit comprising brain areas activated during resting states, and suppressed during cognitive tasks (Anticevic et al., 2012; Fox et al., 2013). Some core DMN areas are also engaged during REM sleep, such as the medial pre-frontal cortex and multiple temporal structures (parahippocampal, hippocampal, and entorhinal cortices; Fox et al., 2013). In patients with schizophrenia, there is an impairment in DMN suppression during attention tasks that may contribute to the cognitive deficits found in these subjects (Anticevic et al., 2012).

The dream experience is also peculiar for psychotic patients. Dream report analysis reveals a higher frequency of nightmares among schizophrenic patients than in healthy subjects (Okorome Mume, 2009; Michels et al., 2014), with more hostile contents, higher proportion of strangers among the dream characters, and a lower frequency of dreams in which the dreamer is the main character (Skancke et al., 2014). We have recently uncovered evidence of language impairments in the dream reports of schizophrenic subjects, who produce substantially less complex narratives than non-schizophrenic subjects (Mota et al., 2014). Using a graph-theoretical approach to represent and quantify word trajectories, we found that the recurrence, connectivity and global complexity of dream reports characterize the distinct patterns of thought disorder that correspond to schizophrenia and bipolar disorder type I, two different diseases associated with psychosis (Mota et al., 2012, 2014). Interestingly, graph connectivity attributes were strongly correlated with negative and cognitive symptoms among psychotic patients (Mota et al., 2014). In other words, psychosis-related cognitive deficits are accompanied by impairment in the ability to share a flow of thoughts when remembering a dream, leading to less connected reports than those produced by healthy subjects. Notably, these differences were more prominent for dream reports than for waking reports (Mota et al., 2014). A likely explanation is the hypo-function of the prefrontal cortex in psychosis, which resembles the reduction of prefrontal cortex activity during REM sleep in healthy subjects, in comparison to the levels found in waking. Both in psychosis and regular dreaming, prefrontal cortex hypo-function seems to be causally related to the decreased criticism typical of these states (Dresler et al., 2014; Laruelle, 2014). Since LD displays increased frontal activity in comparison with non-LD (Mota-Rolim et al., 2010; Stumbrys et al., 2013; Voss et al., 2014), LD has been proposed as potential therapy for psychotic patients (Dresler et al., 2014; Voss et al., 2014).

Despite the large amount of evidence linking sleep and dreaming to psychosis (Gottesmann, 2005; Manoach and Stickgold, 2009; Mota-Rolim and Araujo, 2013; Dresler et al., 2014), there is a lack of quantitative information regarding dreaming in psychotic patients. In particular, there are simply no studies of LD in psychotic patients. To address these gaps, we set out to quantitatively characterize LD in a psychotic sample, using graph-theoretical tools and standard psychiatric instruments to test three hypotheses: (1) Psychotic patients report LD less frequently than non-psychotic subjects; (2) Psychotic patients report LD control less frequently than non-psychotic subjects; and (3) Psychotic patients who experience LD present attenuated psychiatric symptoms and present less thought disorder, in comparison with psychotic patients who do not experience LD.

## Materials and Methods

### Participants

Seventy-three Brazilian individuals (43 males and 22 females, mean age 35.59 ± 10.92 years), comprising 28 subjects without psychotic symptoms (control group – C), 25 patients diagnosed with schizophrenia (S), and 20 patients diagnosed with bipolar disorder type I (B), for a total of 45 medicated patients with psychotic symptoms (**Table 1**). The study was approved by the UFRN Research Ethics Committee (permit#102/06-98244), and the data were collected by convenience sampling at the “Onofre Lopes” and “João Machado” Hospitals. The control group was recruited at the same clinical institutions among subjects presenting anxiety or depression symptoms but without a psychiatric diagnosis (*N* = 11), among psychiatric patients without psychotic symptoms [individuals with depression (*N* = 5), generalized anxiety disorder (*N* = 2), one past episode of post-traumatic stress disorder (*N* = 1)] and healthy individuals accompanying patients (*N* = 9). All individuals gave written informed consent. During the psychiatric interview, patients were examined for major changes in state and level of consciousness (e.g., drowsiness, torpor), for signs of autopsychic and allopsychic disorientation (e.g., inability to remember name, age, spatial localization), and for signs of reduced mnemonic and cognitive capacity. All psychotic subjects were medicated and out of the acute psychotic phase at the onset of the study, so typically they were in good capacity to provide informed consent. When signs of disorientation or reduced mnemonic capacity were detected, the experimenter also obtained written informed consent on their behalf from their legal guardians (next of kin). There were differences related to marital status (more single subject on S than on B, previously married on B than on C and more married subjects on C than on S – which could be explained by social behavior impairments in the psychotic group), medication (more antipsychotics for psychotic groups, more mood stabilizers for B and less antidepressants for S – which reflects the clinical symptoms treated), the age of onset and the duration (smaller age of onset for S compared to C, and larger duration to psychotic group – also expected for the different diseases). Those differences mostly reflect the epidemiological features of a psychotic population within a regular clinical setting.

**Table 1:** Socio-demographic and psychiatric information about the groups investigated.

		Psychotic subjects		Control subjects	*P*-value		
		Schizophrenia	Bipolar		S × B	S × C	B × C
**Demographic characteristics**							
Age	Years	34 ± 9.55	39.05 ± 11.79	34.79 ± 11.25	0.1342	0.8369	0.2910
Sex	Male	84%	65%	61%	0.1406	0.0603	0.7624
	Female	16%	35%	39%			
Education	Years	6.92 ± 4.02	9.35 ± 4.20	8.79 ± 3.94	0.0592	0.0867	0.7232
Marital status	Married	24%	50%	60%	0.0702	0.0071**	0.4607
	Previously Married	20%	30%	8%	0.4380	0.1676	0.0362*
	Never Married	56%	20%	32%	0.0143*	0.0802	0.3507
**Psychiatric assesment**							
Medication	Typical Antipsychotic	72%	65%	0	0.6143	0.0000**	0.0000**
	Atypical Antipsychotic	36%	20%	0	0.2393	0.0027**	0.0350*
	Mood Stabilizer	12%	55%	5%	0.0020**	0.4123	0.0006**
	Benzodiazepine	28%	30%	15%	0.8831	0.2973	0.2560
	Antidepressants	0%	20%	20%	0.0191*	0.0191*	1
Age of onset	Years	22.84 ± 8.27	27.1 ± 9.73	36.8 ± 8.9	0.1013	0.0101*	0.0569
Disease duration	Months	17.32 ± 12.10	12.45 ± 9.98	1.24 ± 1.57	0.2162	0.0011**	0.0042**

Age (years), years of education, frequency of sex, marital status, and medication for the groups studied. Mean and standard deviation are indicated. All subjects were Brazilian. Control subjects were non-psychotic individuals with depression (*N* = 5), generalized anxiety disorder (*N* = 2), one past episode of post-traumatic stress disorder (*N* = 1), various symptoms of mood/anxiety disorder without reaching diagnostic criteria (*N* = 11), plus nine healthy individuals. The groups were compared in pairs using the chi-square test for sex, marital status, and medication, and the Wilcoxon Ranksum test for age, years of education, age of onset, and disease duration. *P*-values are described for each pair comparison (*p < 0.05 and **p < 0.01).

### Instruments

Diagnosis was obtained with SCID DSM IV (First et al., 1990), followed by application of the psychometric scales PANSS (Kay et al., 1987) and BPRS (Bech et al., 1986). We used all the 48 symptoms measured by both scales (30 symptoms measured by PANSS, grades of severity from 1 until 7; and 18 symptoms measured by BPRS, grades of severity from 0 until 3). Next a dream report was requested. Specifically, we asked the subject to report the most recent dream they could remember, followed by questions about regular dreaming (translated from Portuguese: *“Do your dreams usually resemble your daily life?,” “Do your dreams usually resemble your psychotic symptoms?,”* and *“Do your dreams change following changes in medication?”*), and also about LD (*“Can you be aware of dreaming during sleep?*,” *“Can you control your dream when this happens?,” “How frequently does this happen: Once in lifetime, more than once but less than 10 times, more than 10 times but less than 100 times, or more than 100 times?,” “How do you feel when you wake up from these dreams: very good, good, bad or very bad?”*). We considered as lucid dreamers individuals that claimed to be aware of dreaming during a dream at least once in lifetime. All the verbal reports were digitally recorded and transcribed. Analysis: The chi-square test was used to establish statistically significant differences between groups (S, B, and C) on questions about LD, and between lucid dreamers and non-lucid dreamers (within groups S and B) on questions about regular dreams.

### Graph Analysis

Thought disorder was investigated by representing the verbal reports of experimental and control subjects as directed graphs. These were computed by the custom-made free software *SpeechGraphs* (http://www.neuro.ufrn.br/softwares/speechgraphs), which allows the calculation of several attributes related to the recurrence, connectivity, and global complexity of graphs (Mota et al., 2014). This methodology is free of subjective bias, since it does not take into account any personal evaluation of the semantic content of the verbal reports. Rather, it mathematically analyzes various structural aspects of the reports. We have previously validated this methodology for the diagnosis of psychosis (Mota et al., 2012, 2014) and dementia (Bertola et al., 2014). The rationale for combining the use of psychometric scales and speech graph analysis was to quantitatively analyze the psychiatric symptoms, so as to compare groups of lucid and non-lucid psychotic dreamers and better characterize their mental functioning. A graph is a mathematical representation of a network with nodes linked by edges, formally defined as *G* = (*N*, *E*), with the set of nodes *N* = {*w*_*1*_, *w*_*2*_, …, *w*_*n*_} and the set of edges *E* = {(*w*_*i*_,*w*_*j*_)} (Mota et al., 2012, 2014; Bertola et al., 2014). A speech graph represents the sequential relationship of spoken words in a verbal report, with each word represented as a node, and the sequence between successive words represented as a directed edge (Mota et al., 2012, 2014; Bertola et al., 2014). A total of 14 speech graph attributes (SGA) were calculated for each dream report, comprising general graph attributes (*N*, total of nodes; *E*, total of edges), recurrence (PE, parallel edges; RE, repeated edges; L1, L2, and L3, loops of one; two and three nodes), connectivity (LCC, largest connected component and LSC, largest strongly connected component) and global attributes (ATD, average total degree; Density, Diameter; ASP, average shortest path; CC, clustering coefficient; **Table 2**).

**Table 2:** Speech graph attributes (SGA): detail description of each speech graph attribute measured from dream reports.

**N:** Number of nodes.
**E:** Number of edges.
**RE (Repeated Edges):** sum of all edges linking the same pair of nodes.
**PE (Parallel Edges):** sum of all parallel edges linking the same pair of nodes given that the source node of an edge is the target node of the parallel edge.
**L1 (Loop of one node):** sum of all edges linking a node with itself, calculated as the trace of the adjacency matrix.
**L2 (Loop of two nodes):** sum of all loops containing two nodes, calculated by the trace of the squared adjacency matrix divided by two.
**L3 (Loop of three nodes):** sum of all loops containing three nodes (triangles), calculated by the trace of the cubed adjacency matrix divided by three.
**LCC (Largest Connected Component):** number of nodes in the maximal subgraph in which all pairs of nodes are reachable from one another in the underlying undirected subgraph.
**LSC (Largest Strongly Connected Component):** number of nodes in the maximal subgraph in which all pairs of nodes are reachable from one another in the directed subgraph (node a reaches node b, and b reaches a).
**ATD (Average Total Degree):** given a node n, the Total Degree is the sum of “in and out” edges. Average Total Degree is the sum of Total Degree of all nodes divided by the number of nodes.
**Density:** number of edges divided by possible edges. [*D* = 2*E/N*(*N* – 1)], where *E* is the number of edges and *N* is the number of nodes.
**Diameter:** length of the longest shortest path between the node pairs of a network.
**Average Shortest Path (ASP):** average length of the shortest path between pairs of nodes of a network.
**CC (Average Clustering Coefficient):** given a node n, the Clustering Coefficient Map (CCMap) is the set of fractions of all n neighbors that are also neighbors of each other. Average CC is the sum of the Clustering Coefficients of all nodes in the CCMap divided by number of elements in the CCMap.

The non-parametric statistical test Wilcoxon Ranksum was used to establish SGA differences between lucid dreamers and non-lucid dreamers, as well as differences in the symptomatology measured by psychometric scales and speech measures (corrected for the number of symptoms and speech attributes by the Bonferroni method, α = 0.0008). Effect size was measured by Cohen’s *d*.

## Results

**FIGURE 1:**
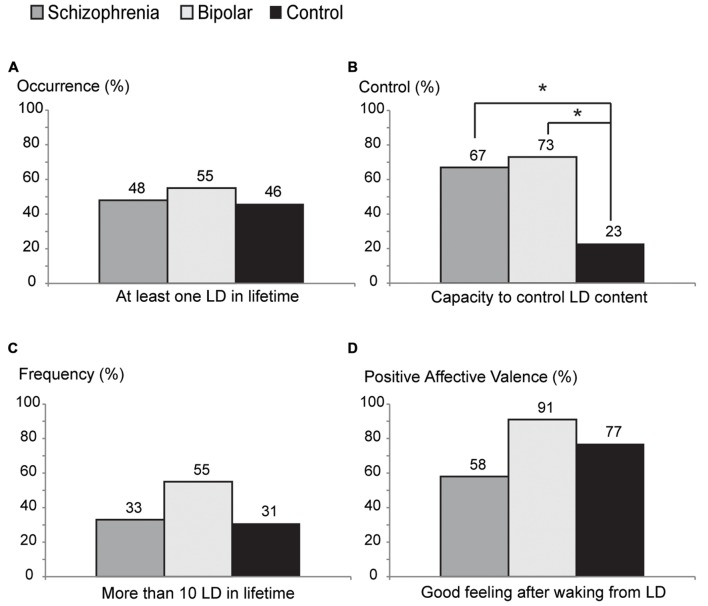
**Characteristics of lucid dream reports in schizophrenia (S), bipolar (B), and control (C) groups. (A)** Percentage of each group reporting occurrence of lucid dreaming at least once in a lifetime. **(B)** Percentage of the ability to control their dreams: psychotic groups report control ability more frequently than control group (S vs. C: *p* = 0.0283, B vs. C: *p* = 0.0150). **(C)** Percentage of high frequency of lucid dreams (more than 10 lucid dreams in a lifetime). **(D)** Percentage of positive affective valence (good feeling after wake up from a lucid dream) (*p < 0.05).

About half of the psychotic subjects (48% of S and 55% of B) and 46% of C reported having at least one LD in life, but we found no statistically significant difference among the groups S versus B (*p* = 0.6407), S versus C (*p* = 0.3138), or B versus C (*p* = 0.5582; **Figure 1A**). Psychotic lucid dreamers reported control of their dreams more frequently (67% of S and 73% of B) than non-psychotic lucid dreamers (only 23% of C; S versus C *p* = 0.0283, B versus C *p* = 0.0150; **Figure 1B**). There was no statistical difference among groups concerning the number of lifetime LD episodes (33% of S, 55% of B, and 31% of C reported having had more than 10 LD in life; S versus B *p* = 0.3053, S versus C *p* = 0.8908, B versus C *p* = 0.2391; **Figure 1C**), nor for the proportion of subjects that reported feeling good after waking up from a LD (58% of S, 91% of B, and 77% of C; S versus B *p* = 0.0755, S versus C *p* = 0.3195, B versus C *p* = 0.3596; **Figure 1D**). Specifically regarding lucid dreamers in the psychotic groups, 57% of those that were unable to control LD, and 81% of those that claimed to control LD, reported pleasant feelings after waking from a LD (no statistical difference between lucid dreamers that control the dream and lucid dreamers that do not control the dream on S and B groups, *p* = 0.2257).

A possible confounding factor to interpret the higher fre quency of dream control in the psychotic groups is the use of antipsychotic medications. Neurons in the prefrontal cortex are among the main targets of antipsychotics, via modulation of the prefrontal cortex output to basal ganglia circuits (Monti and Monti, 2004; Merikangas et al., 2011). First generation antipsychotics enhance total sleep time and sleep efficiency by controlling psychotic symptoms, but there are no consistent results in non-psychotic subjects. Second generation antipsychotics increase total sleep time and sleep efficiency in both psychotic and non-psychotic subjects, with some drugs having specific effects on sleep patterns (e.g., olanzapine increases the amount of the N2 stage of sleep; Monti and Monti, 2004; Cohrs, 2008). To investigate this effect in our psychotic sample, we compared the doses of antipsychotics (chlorpromazine-equivalent) between lucid and non-lucid dreamers. Within lucid dreamers, we compared the antipsychotic doses administered to those that reported to control LD to the doses administered to those who reported not to control their dreams. Neither comparison showed statistically significant differences (lucid versus non-lucid dreamers *p* = 0.5460, and control versus non-control *p* = 0.8556), thus strengthening the conclusion that the differences between psychotic and control groups concerning the ability to control LD are related to the psychotic state, not to the different medications used.

**FIGURE 2:**
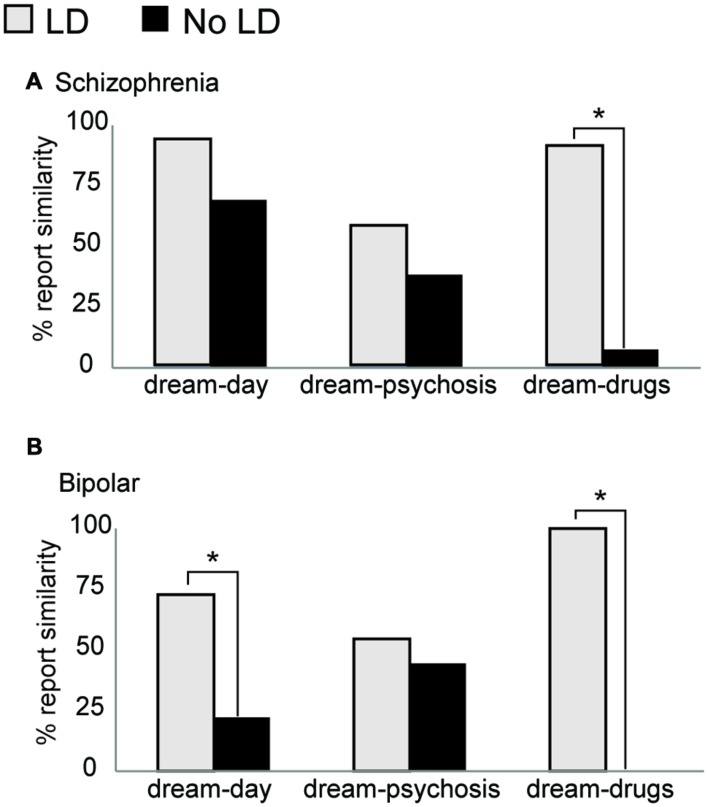
**Characteristics of regular dream reports among psychotic patients. (A)** Within the S group, there were no significant differences between lucid dreamers and non-lucid dreamers concerning similarities between dream and daily experiences, but lucid dreamers reported changes on dream contents after changes on medication more frequently than non-lucid dreamers (*p* < 0.00005). **(B)** In the B group, lucid dreamers reported similarities between dream and daily experiences, as well as changes on dreams after medication changes, more frequently than non-lucid dreamers (*p* = 0.0246 and *p* < 0.00005, respectively). Neither S nor B showed differences between lucid and non-lucid dreamers on reports about similarities between dreams and psychotic symptoms (*p < 0.05).

Among psychotic patients, lucid dreamers reported similarities between dreams and daily life more frequently than non-lucid dreamers (for B: 73% of lucid dreamers and 22% of non-lucid dreamers, *p* = 0.0246; for S: 94% of lucid dreamers and 69% of non-lucid dreamers, *p* = 0.0596; **Figure 2**). Following changes in medication, lucid dreamers were much more likely to report changes in dream content (100% of B and 92% of S) than non-lucid dreamers (0% of B, and 8% of S; *p* = 0.0000 on S and B; **Figure 2**). **Figure 2** also shows that there was no difference concerning the similarity of dreams and symptoms between lucid (55% of B, and 58% of S) and non-lucid (44% of B, and 38% of S; *p* = 0.3204 on S and *p* = 0.6531 on B) dreamers.

**FIGURE 3:**
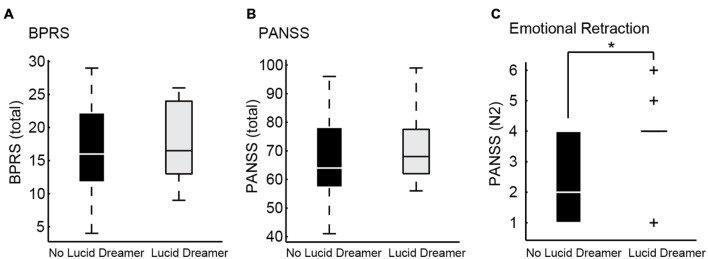
**Psychometric differences between lucid dreamers and non-lucid dreamers among schizophrenia patients. (A)** Boxplots showing total BPRS of lucid dreamers and non-lucid dreamers in the S group (*p* = 0.5930). **(B)** Boxplots showing total PANSS of lucid dreamers and non-lucid dreamers in the S group (*p* = 0.6434). **(C)** Among S subjects, lucid dreamers showed higher scores on PANSS item N2 about emotional retraction (*p* = 0.0329), without significant differences for the other symptoms; no significant differences were found among B subjects (see Supplementary Table 1) (*p < 0.05).

**FIGURE 4:**
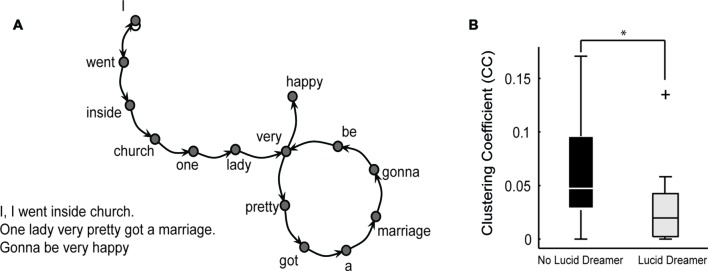
**Differences on speech structure when reporting a regular dream between lucid dreamers and non-lucid dreamers among schizophrenia patients. (A)** Example of a text (regular dream report) represented as a speech graph. For this plot the original text was in Portuguese and each word was translated to English, preserving the original grammatical structure. Speech graph attributes (SGA, see **Table 2**) were used to characterize speech structure from dream reports. **(B)** In the S group, speech graphs from dream reports of lucid dreamers showed smaller clustering coefficient (CC) than non-lucid dreamers (*p* = 0.0171) (*p < 0.05).

With regard to the application of standard psychometric scales and speech quantitative analysis, we did not find any difference between lucid and non-lucid dreamer patients, neither in S nor in B groups after correction for multiple comparisons (α = 0.0008). We failed to detect any clinical advantage for lucid dreamers even when multiple comparisons were disregarded (α = 0.05), even for the item G12 on PANSS, related to the symptom “Lack of judgment and insight.” This means that the psychotic patients that were more able to have insight during dreaming were not more able to have insight about their own psychotic reality than patients that were less aware during dreaming. On the contrary, the emotional retraction symptom measured by item N2 of the PANSS Negative Subscale, (Kay et al., 1987) was more prevalent in lucid dreamers than in non-lucid dreamers among S [**Figure 3** and Supplementary Table 1; LD versus non-LD on S: *p* = 0.0329, mean ± SD non-lucid (*n* = 13): 2.54 ± 1.28 lucid (*n* = 12): 3.75 ± 1.36; Cohen’s *d*: –0.92, a large effect size]. This symptom is characterized by the lack of interest in external events, with little involvement or affective commitment. Likewise, with regard to the structural features of speech, only in S we found that lucid dreamers displayed a significantly different SGA, namely smaller clustering coefficient [CC; *p* = 0.0171, mean ± SD non-lucid (*n* = 13): 0.065 ± 0.047 lucid (*n* = 12): 0.030 ± 0.037; Cohen’s *d*: 0.83, a large effect size] in comparison with non-lucid dreamers (**Figure 4** and Supplementary Table 2). This means that lucid dreamers in the S group produced less complex speech graphs when reporting a regular dream, in comparison with S subjects that were not lucid dreamers, reflecting a less complex flow of thought.

## Discussion

Altogether, the results falsified the three hypotheses that we set out to test. First, psychotic patients did not report LD less frequently than non-psychotic subjects. Second, among the subjects that reported being lucid dreamers, psychotic patients reported LD control more frequently than non-psychotic subjects. Finally, patients who reported LD did not present attenuated psychiatric symptoms, in comparison with patients who did not report LD. Indeed, schizophrenia patients that qualified as lucid dreamers showed a tendency to be more, not less symptomatic than non-lucid dreamers in the same group. Therefore, although the results on the lifetime occurrence of LD replicate prior data (Snyder and Gackenbach, 1988; Mota-Rolim et al., 2013), we could not find support for the notion that a psychotic sample would report less LD than a non-psychotic sample. There was no difference between psychotic and non-psychotic subjects regarding the number of LD events in life. As previously detected in a non-psychotic sample (Voss et al., 2013), we found positive emotions to be more frequently associated with LD in all groups, without significant differences.

In a sample of 3,427 Brazilian subjects interviewed online, 29% of the subjects reported the ability to control LD (Mota-Rolim et al., 2013). In the present study, only 23% of the non-psychotic sample reported LD control, in contrast with significantly larger numbers among psychotic subjects (67% of S and 73% of B). This result was unexpected, considering that non-psychotic lucid dreamers show increased control of internal reality (Blagrove and Tucker, 1994; Blagrove and Hartnell, 2000), being more frequently able to regulate cognition and emotion than non-lucid dreamers (Blagrove and Hartnell, 2000). A possible explanation is that psychosis enhances the experience of the internal reality in detriment of the external reality, and therefore lucid dreamers with psychotic symptoms would be more able to control their internal reality than non-psychotic lucid dreamers. If we hypothesize that the positive symptoms of psychosis may represent the intrusion of REM sleep mentation into waking (Freud, 1900; Dzirasa et al., 2006; Dresler et al., 2014), and that LD may reflect the intrusion of waking mentation into REM sleep (Mota-Rolim and Araujo, 2013), subjects who frequently experience both conditions may be more cognitively trained to control their internal reality than those who rarely experience LD. This line of reasoning is supported by the fact that lucid dreamers with psychotic symptoms reported more similarity between dreams and daily life than non-lucid dreamers with psychotic symptoms. Lucid dreamers were also much more likely than non-lucid dreamers to report changes in dream content following changes in medication, possibly reflecting a higher awareness of dream reality in the former. Indeed, the frequent experience of REM sleep-like mentations into the waking life might train control of internal reality, and thus explain higher control of lucid dream in psychotic patients. This might be particularly true for transition phases between acutely psychotic and non-psychotic phases. Within the dreaming/psychosis model, such transition phases might thus be considered as “pre-lucid.” Future studies should consider a longitudinal design, and aim to characterize the transition between acute and non-acute psychotic phases.

We found no clinical advantages of having LD with regard to psychiatric symptomatology, to speech structure, and in particular to criticism of reality [question G12 of PANSS (Kay et al., 1987), Supplementary Table 1]. On the contrary, we found that lucid dreamers in the S group tends to be more emotionally retracted than non-lucid dreamers, which means that they were more isolated from others. These subjects also tended to report their regular dreams in a less clustered manner, reflecting a decrease in the complexity of the flow of thought when reporting a dream, a symptom related to cognitive and negative severity in schizophrenia (Mota et al., 2014), and with cognitive impairment in dementia (Bertola et al., 2014). Although these results do not reach significance after Bonferroni correction, they have a large effect size that should not be neglected. Possibly if the number of subjects per group was higher, these symptomatology differences would become clearer. Taken together, both psychometric features reveal impairment of social behavior and thought disorganization among lucid dreamers in the S group, which could be considered a potential disadvantage related to clinical severity. But considering that those lucid dreamers tend to control dream contents more frequently, we can also interpret this result as a compensatory attempt to enhance dream control, rather than trying the more difficult control of reality. Do changes in dream control precede changes in reality control, or vice-versa? While the transversal design employed here cannot answer this question, future longitudinal studies should help to disentangle these alternatives, by synchronously collecting data on insights about dreaming and psychotic reality, to determine the order of occurrence of changes in these states.

Our study has other limitations that need to be considered. First, sample sizes were relatively small, reflecting the scarcity of individuals that experience both psychotic symptoms and LD. The prevalence of LD (considering the definition adopted in this study) is high in the Brazilian population (77.2%; Mota-Rolim et al., 2013) and was not found to be low in our sample (48% in S, 55% in B, and 46% in C), but the prevalence of psychosis is much lower (B prevalence data from 11 countries: 0.6%; Merikangas et al., 2011, S prevalence data from 46 countries: 0.55%; McGrath et al., 2008). We also had differences between the groups that mostly reflect general epidemiological differences regarding marital status within psychotic populations, but should be considered as a potential confounding factor. In addition, the control sample (subjects without psychotic symptoms in lifetime) had a mixture of individuals with and without psychiatric symptoms, some with psychiatric diagnosis like depression and others without any psychiatric symptom in lifetime, what make this control group very heterogeneous; in future studies a control sample without any psychiatric symptoms should be investigated.

Another caveat is the fact that the research was only based on self-reports of LD, with possible confounds of secondary elaboration, motivation, conscious and unconscious intentions (Freud, 1900). Ideally lucidity should be assessed by external judges to avoid fallacious interpretations (Stumbrys et al., 2012). Moreover, we assessed LD throughout the lifetime, but did not investigate whether the patients experienced lucid dreams specifically during the psychotic episode(s). This is an important issue to be clarified in future studies, specifically when considering symptomatology differences, such as the increase of insight. Maybe the patients that were considered as lucid dreamers in the present study were not experiencing lucid dreams during that period, and would not show potential clinical advantages such as increased insight.

Medication was another limitation to consider (**Table 1**), since all the psychotic subjects were medicated with a variety of different drugs, and the use of psychotropic drugs can modify dream perception and recall (Solms, 2000; Gottesmann, 2005). Future studies should also interview psychotic patients during acute crises, to compare with the data collected during non-acute states as in the present study. In principle, data sampled during acute phases should be more informative. The symptomatology during this transition phase (acute to non-acute phase) should give important information regarding changes in insight of the differences between fantasy and reality.

Furthermore, we did not control for differences in dream recall frequency among the patients, an important methodo logical issue for dream research (Schredl, 2011; Michels et al., 2014; Skancke et al., 2014), which could perhaps explain the differences in continuity between daily life and dreams, or changes of dream content after change of medication. In addition, we did not control for differences in the frequency of nightmares, which is heightened in S patients (Okorome Mume, 2009; Michels et al., 2014; Skancke et al., 2014), and may be related with lucidity in pathological conditions (Rak et al., 2015). However, nightmares are by definition associated with unpleasant feelings after waking up, and we found a high frequency of pleasant feelings after waking up from a lucid dream in this sample (58% for S and 91% for B). Finally, we did not employ training or induction techniques for LD generation (Stumbrys et al., 2012), but rather dealt with natural recollections of spontaneous LD. The results in trained subjects may be quite different from those reported here. Beyond these limitations, our results suggest that psychotic lucid dreamers, which fail the “external reality test,” are nevertheless more able to control their internal reality during dreaming.

To the best of our knowledge, the present study is the first to assess LD in a clinically characterized psychotic sample. Overall the results confirm the notion that LD is associated with psychosis. This relationship deserves a closer investigation, since the present data does not conform to the hypothesis that LD control is helpful to psychotic patients. The distinctive features of the LD experience in our sample pose a challenge to the perspective of clinically using LD for the treatment of psychosis (Dresler et al., 2014; Voss et al., 2014). Also, the results point to an intriguing relationship between dream lucidity and judgment of reality among psychotic patients, which deserves deeper investigation with larger samples. Training dream lucidity is likely to produce safe psychological strengthening in a non-psychotic population (Stumbrys et al., 2012), but in a psychotic population LD practice may further empower deliria and hallucinations, giving internal reality the appearance of external reality.

## Author Contributions

NM and SR designed the study, collected the data, NM, AR, SM-R, MC, and SR analyzed the data, and NB, SR, SM-R, and MC wrote the paper.

## Conflict of Interest Statement

The authors declare that the research was conducted in the absence of any commercial or financial relationships that could be construed as a potential conflict of interest.
