# Supplementary material for: Psychosis and the Control of Lucid Dreaming
Source: Front Psychol. 2016 Mar 9;7:294. doi: 10.3389/fpsyg.2016.00294 (PMC4783408; doi:10.3389/fpsyg.2016.00294)
Supplement: Supplementary file 1 [file Image_1.PDF]

Supplementary Table 1: Comparison of psychometric assessments on psychotic patients that claim to had lucid dreams at least once in lifetime versus patients that never had lucid dream. Psychometric scales (PANSS and BPRS) differences between lucid dreamers (LD) and non-lucid dreamers (No LD) on groups Schizophrenia and Bipolar (p values using Wilcoxon Rank-sum test, significant results in red).

SCHIZOPHRENIA	LD x No LD (p value)	BIPOLAR	LD x No LD (p value)
PANSS		PANSS	
Total	0.5930	Total	0.3419
Positive	0.6599	Positive	0.2534
Negative	0.2953	Negative	0.6428
General	1	General	0.1708
P1	0.4600	P1	0.3099
P2	0.8014	P2	0.6915
P3	0.3851	P3	0.9039
P4	0.7784	P4	0.5309
P5	0.1993	P5	0.6076
P6	0.8600	P6	0.3731
P7	0.3601	P7	0.8695
N1	0.3470	N1	0.8226
N2	0.0329	N2	0.2153
N3	0.2853	N3	0.8703
N4	0.3013	N4	0.7479
N5	0.8984	N5	0.6050
N6	0.4972	N6	0.4214
N7	0.2404	N7	0.5795
G1	1	G1	0.3667
G2	0.4370	G2	0.3518
G3	0.4166	G3	0.6502
G4	0.2543	G4	0.2390
G5	0.6158	G5	0.4214
G6	0.7611	G6	0.4952
G7	0.6116	G7	0.4190
G8	0.2958	G8	0.5011
G9	0.8393	G9	0.5732
G10	0.8619	G10	0.2409
G11	0.7068	G11	0.2764
G12	0.8776	G12	0.7439
G13	0.6218	G13	0.7781
G14	0.2427	G14	0.5150
G15	0.6689	G15	0.8925
G16	0.4996	G16	0.7533
BRPS		BPRS	
Total	0.6434	Total	0.3603
1	0.7983	1	0.3878
2	0.1725	2	0.3813
3	0.1037	3	0.7914
4	0.5133	4	0.8655
5	0.4476	5	0.8331
6	0.4957	6	0.1447
7	0.3668	7	1
8	0.3109	8	0.9682
9	0.8753	9	0.9020
10	0.2427	10	0.8695
11	0.7795	11	1
12	0.7633	12	1
13	0.8372	13	0.8806
14	0.5418	14	0.5408
15	0.4330	15	0.3380
16	0.6680	16	0.2247
17	0.3326	17	0.6058
18	0.7389	18	0.2111
