# Supplementary material for: Psychosis and the Control of Lucid Dreaming
Source: Front Psychol. 2016 Mar 9;7:294. doi: 10.3389/fpsyg.2016.00294 (PMC4783408; doi:10.3389/fpsyg.2016.00294)
Supplement: Supplementary file 2 [file Image_2.PDF]

Supplementary Table 2: Comparison of speech graph attributes or SGAs (Supplementary File 1) on psychotic patients that claim to had lucid dreams at least once in lifetime versus patients that never had lucid dream. Speech graph attribute (SGA) differences between lucid dreamers (LD) and non-lucid dreamers (No LD) on groups Schizophrenia and Bipolar (p values using Wilcoxon Ranksum test, significant results in red).

SCHIZOPHRENIA	LD x No LD	BIPOLAR	LD x No LD
SGA		SGA	
Nodes	0.5767	Nodes	0.8792
Edges	0.4360	Edges	0.5433
RE	0.8651	RE	0.8792
PE	0.6447	PE	0.9394
L1	0.8407	L1	0.2388
L2	0.2914	L2	0.4940
L3	0.0701	L3	0.6485
LCC	0.7158	LCC	0.1965
LSC	0.7158	LSC	0.1965
ATD	0.4518	ATD	1
Density	0.2540	Density	1
Diameter	0.9034	Diameter	0.8197
ASP	0.9420	ASP	0.8792
CC	0.0171	CC	0.7612
